# Caveolin-1 Deficiency Leads to Increased Susceptibility to Cell Death and Fibrosis in White Adipose Tissue: Characterization of a Lipodystrophic Model

**DOI:** 10.1371/journal.pone.0046242

**Published:** 2012-09-26

**Authors:** Sally Martin, Manuel A. Fernandez-Rojo, Amanda C. Stanley, Michele Bastiani, Satomi Okano, Susan J. Nixon, Gethin Thomas, Jennifer L. Stow, Robert G. Parton

**Affiliations:** 1 Institute for Molecular Bioscience, The University of Queensland, Brisbane, Queensland, Australia; 2 Centre for Microscopy and Microanalysis, The University of Queensland, Brisbane, Queensland, Australia; 3 University of Queensland Diamantina Institute, The University of Queensland, Brisbane, Queensland, Australia; Ecole Polytechnique Federale de Lausanne, Switzerland

## Abstract

Caveolin-1 (CAV1) is an important regulator of adipose tissue homeostasis. In the present study we examined the impact of CAV1 deficiency on the properties of mouse adipose tissue both *in vivo* and in explant cultures during conditions of metabolic stress. In CAV1^−/−^ mice fasting caused loss of adipose tissue mass despite a lack of hormone-sensitive lipase (HSL) phosphorylation. In addition, fasting resulted in increased macrophage infiltration, enhanced deposition of collagen, and a reduction in the level of the lipid droplet protein perilipin A (PLIN1a). Explant cultures of CAV1^−/−^ adipose tissue also showed a loss of PLIN1a during culture, enhanced secretion of IL-6, increased release of lactate dehydrogenase, and demonstrated increased susceptibility to cell death upon collagenase treatment. Attenuated PKA-mediated signaling to HSL, loss of PLIN1a and increased secretion of IL-6 were also observed in adipose tissue explants of CAV1^+/+^ mice with diet-induced obesity. Together these results suggest that while alterations in adipocyte lipid droplet biology support adipose tissue metabolism in the absence of PKA-mediated pro-lipolytic signaling in CAV1^−/−^ mice, the tissue is intrinsically unstable resulting in increased susceptibility to cell death, which we suggest underlies the development of fibrosis and inflammation during periods of metabolic stress.

## Introduction

Dysregulation of systemic lipid levels plays an important role in the development of numerous metabolic disorders including obesity and lipodystrophy [1], [2], [3]. Adipose tissue is central to lipid regulation, facilitating both the storage of fatty acids as neutral lipids within the lipid droplets (LDs) of adipocytes, and regulating the release of fatty acids in response to both acute and chronic stimuli. In metabolic disorders these essential functions of adipose tissue are compromised. Determining the cellular mechanisms underlying the dysregulation of adipocytes is fundamentally important to understanding adipose tissue regulation and metabolism.

The mobilization of fatty acids from adipose tissue *in vivo* is regulated by specific mechanisms (reviewed in [4]). Catecholamines acutely stimulate lipolysis through the activation of beta-adrenergic receptors at the adipocyte cell surface (reviewed in [5], [6]). This results in the activation of a well characterized cAMP-dependent, G-protein coupled signal transduction cascade culminating with the phosphorylation and activation of proteins at the surface of LDs by protein kinase A (PKA), including the major structural protein in the adipocyte LD, perilipin A (PLIN1a) [7], and the primary diacylglycerol (DAG) lipase, hormone-sensitive lipase (HSL) [8], [9]. During fasting the mobilization of fatty acids can be chronically activated through a combination of increased adrenaline and glucagon and reduced levels of insulin [10]. In addition cytokines such as tumor necrosis factor (TNF) and interleukin-6 (IL-6) have also been shown to promote lipolysis both *in vitro* and *in vivo* [11].

Caveolin-1 (CAV1) is an integral membrane protein that together with members of the Cavin family of proteins is essential for the formation of caveolae at the cell surface [12], [13]. Caveolae are highly abundant organelles in adipocytes comprising up to 30% of the cell surface [14], [15]. Despite numerous studies the precise role of CAV1 and caveolae in the regulation of lipid metabolism remains elusive (reviewed in [16]). In model adipocyte cell lines loss of Cavin-1 attenuates stimulated glycerol release suggesting an important role of caveolae in promoting lipolysis [17]. Consistent with this, CAV1 knockout mice (CAV1^−/−^) show impaired catecholamine-stimulated lipolysis and PKA signaling in adipose tissue [18], [19]. However, differentiated MEFs from CAV1^−/−^ mice show enhanced stimulated lipolysis and normal pro-lipolytic signaling [20], suggesting that some of the effects observed *in vivo* might be secondary effects, rather than primary defects, due to loss of the proposed CAV1 scaffold [18]. Furthermore, CAV1^−/−^ mice are resistant to diet-induced obesity and show a mild lipodystrophy [18], [21], [22], [23], and human mutations in CAV1 are associated with a severe lipodystrophic phenotype [24], suggesting defects in lipid storage, adipogenesis or in adipose tissue homeostasis. Finally, stored triglyceride in brown adipose tissue from CAV1^−/−^ mice is metabolized normally for thermogenesis [25] despite the loss of catecholamine stimulation [19], while fasting induces a loss of body weight and adipose tissue mass in CAV1^−/−^ mice, concomitant with an increase in serum free fatty acids, suggesting a normal metabolic response to fasting [21]. Together these data suggest that CAV1 and caveolae play pleiotropic roles in adipose tissue regulation and function. These roles are likely to include general regulatory mechanisms such as signaling and lipid transfer, together with context specific roles related to the adipose tissue microenvironment or specific metabolic challenges.

In the current study we have examined adipose tissue from CAV1^−/−^ mice both during fasting and following maintenance on a high fat diet. Fasting caused loss of adipose tissue, despite a loss of PKA-mediated site-specific HSL phosphorylation, increased macrophage infiltration into adipose tissue, enhanced deposition of collagen, and a reduction in the level of the lipid droplet protein PLIN1a. Loss of PLIN1a could be recapitulated by *ex vivo* culture of CAV1^−/−^ adipose tissue, which correlated with enhanced secretion of IL-6 and release of lactate dehydrogenase. Consistent with structural fragility of CAV1^−/−^ adipocytes, collagenase treatment of adipose tissue resulted in significantly increased rates of cell death relative to tissue from control mice. Together these results suggest that CAV1 loss from adipose tissue affects adipocyte robustness, resulting in increased collagen deposition and eliciting an inflammatory response. Intriguingly, the phenotype of adipose tissue in CAV1^−/−^ mice closely mirrored that of wild type mice maintained on a high fat diet, but without the increase in obesity, suggesting that the inflammatory state observed in obesity could be directly associated with alterations in the adipocyte cell surface. Intrinsic tissue instability could impact on both the development of an inflammatory state and, in the case of CAV1 deficiency, the development of lipodystrophy.

## Materials and Methods

### Antibodies and Reagents

Rabbit anti-Phospho PKA Substrate (RRxS*/T*) (#9624), P-HSL^Ser563^ (#4139), P-HSL^Ser660^ (#4126) and HSL (#4107) were obtained from Cell Signaling Technology, rabbit anti-PLIN1a (P1998) was from Sigma (St Louis, Missouri), mouse anti-beta actin antibody (#MAB1501) was from Chemicon, rat anti-mouse F4/80 was from Serotec (Oxford, UK), mouse anti-PKAcat (#610980) was from BD Biosciences and mouse anti-collagen-1 was from Abcam (ab34710). Forskolin was obtained from Merck (Merck Pty Ltd, Kilsyth, Australia). Glycerol release was measured using free glycerol reagent (Sigma, St Louis, USA). The release of non-esterified fatty acid (NEFA) was measured using the NEFA kit from Wako Pure Chemicals Industries Ltd (Osaka, Japan). Collagenase I was obtained from Worthington Biochemical Corp. (Lakewood, NJ, USA). Remaining reagents were obtained from Sigma unless stated otherwise.

### Animal Studies

CAV1^−/−^ mice were developed in the Kurzchalia laboratory [26]. CAV1^−/−^ and CAV1^+/+^ mice were kept under a controlled humidity and lighting schedule with a 12 h dark period. All animals received care in strict compliance with institutional guidelines regulated by the Australian Government. All animal experiments were performed with the approval of the University of Queensland Animal Ethics Committee. Food and water were available *ad libitum*. ***Fasting***. CAV1^−/−^ and CAV1^+/+^ mice (male, 10–18 weeks old) were used. Food withdrawal was initiated at the start of the light period and fasting continued for the times shown in the results section. ***High fat diet*** *.* CAV1^−/−^ and CAV1^+/+^ mice (male, 10–14 weeks old) were fasted for 24 hours then fed *ad libitum* with control diet (Research diets, # D12450B, 10% kcal% fat) or a high fat diet (Research diets, # D12492, 60% kcal% fat) for 12 weeks. At the end of the diet period mice were sacrificed, samples of plasma obtained and adipose tissue immediately frozen in liquid nitrogen and stored at −80°C.

### Mouse Adipose Tissue *ex vivo* Culture

Adipose tissue was obtained from the epididymal fat pads of CAV1^+/+^ and CAV1^−/−^ mice between the ages of 12–21 weeks. Explants were cultured as described previously [27]. Collagenase digestion was performed as described previously [28].

### Cytokine Analysis

Blood was extracted by cardiac puncture and collected in BD microcontainer PST LH (#365987). Serum was collected after centrifugation of blood samples at 6000 rpm for 10 min at RT. Explant media samples were collected directly from the culture vessels. Cytokine levels were measured in serum and media samples using Flex Set bead array kits and a FACSCanto II (BD Biosciences), according to the manufacturers instructions.

### Body Weight and Dual-energy X-ray Absorptiometry (DXA) Analysis

To study the effect of fasting on body and adipose tissue mass we measured both the weight and the % body fat of CAV1^+/+^ and CAV1^−/−^ mice. Body fat was measured using DXA. Mice were anaesthetized with Ketamine (80 mg/kg) and Xylazine (10 mg/kg) administered ip and scanned within a PIXImus Densitometer (GE Medical Systems) to measure % body fat prior to and immediately following a 24 h fast.

### Sample Preparation and Western Blotting

Tissue or explants were homogenized in TNE (10 mM Tris-HCl, pH 7.4, 150 mM NaCl, 5 mM EDTA) containing protease (Merck Pty Ltd, Kilsyth, Australia) and phosphatase (Roche Diagnostics Australia, Castle Hill, Australia) inhibitors using an Ultra-Turrax homogenizer (IKA, Kuala Lumpur, Malaysia). SDS-PAGE and Western blot analysis was carried out as described previously [27]. Relative band intensity was measured using the Image J software and corrected for background using an equivalent area of blank film.

### Immunohistochemistry of Adipose Tissue

Epididymal adipose tissue from ∼14 week old CAV1^−/−^ and CAV1^+/+^ mice was fixed in 4% PFA in PBS. Tissue was processed and embedded in paraffin by the QIMR Histology service. Sections (4 µm) were stained with Sirius red to detect collagen (QIMR Histology Service). To detect macrophages sections were cleared and hydrated using standard methods, digested using the Carezyme-1 Trypsin (Biocare Medical, CA, USA) and labelled for F4/80 using the R.T.U. Vectastain Elite ABC kit according to manufacturers instructions (Vector laboratories, CA, USA). Reaction product was visualized by incubating with ImmPACT DAB substrate (Vector laboratories). Images were taken using a DP-70 12 Mp colour camera mounted on an Olympus BX-51 microscope.

## Results

### Fasting Results in an Equivalent Loss of Adipose Tissue Despite Attenuated PKA-Mediated HSL Phosphorylation in CAV1^−/−^ Mice

**Figure 1:**
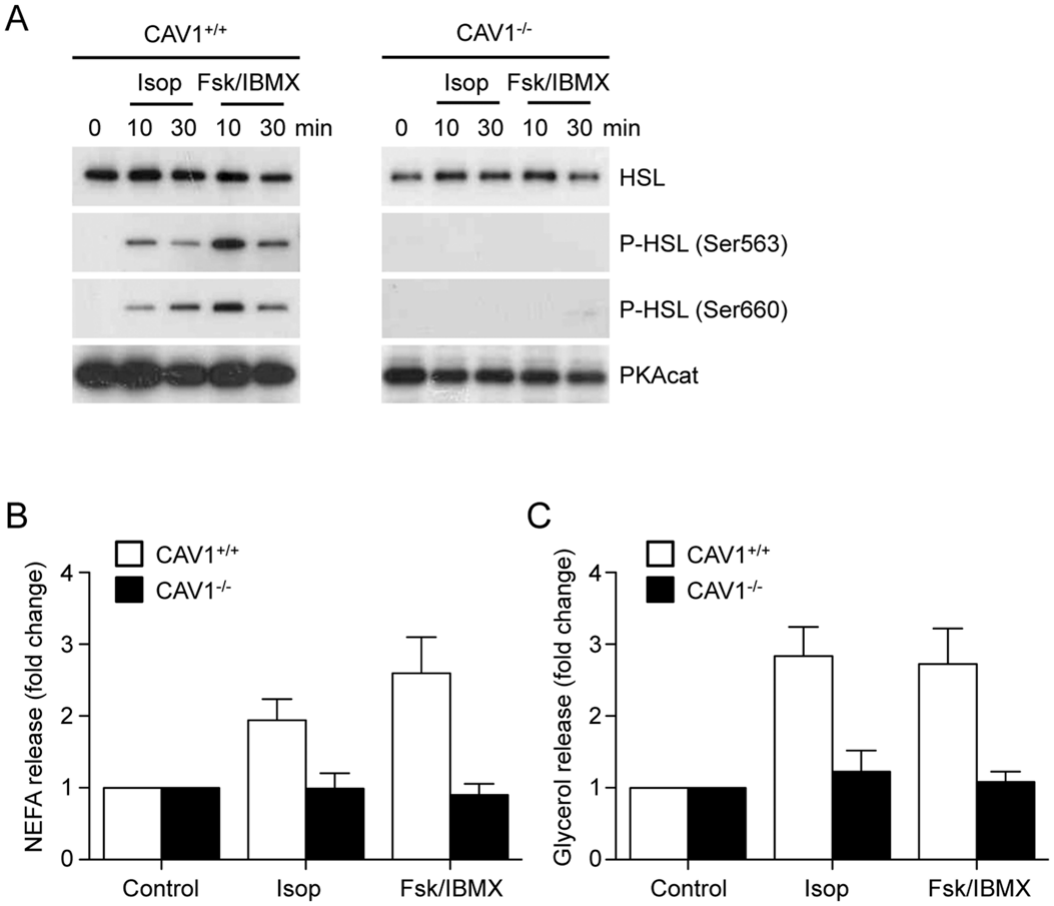
Activation of lipolysis in epididymal adipose tissue explants from CAV1^+/+^ and CAV1^−/−^ mice. Adipose tissue explants were cultured from epididymal adipose tissue of CAV1^−/−^ and CAV1^+/+^ mice and lipolysis activated using the beta-adrenergic agonist isoproterenol (Isop, 10 µM) or by direct elevation of cAMP levels using forskolin (10 µM) and IBMX (500 µM) (Fsk/IBMX). In CAV1^−/−^ explants neither activation regime resulted in the PKA-mediated phosphorylation of HSL on either Ser563 or Ser660, despite the expression of the PKA catalytic subunit (**A**), or stimulated the release of (**B**) glycerol or (**C**) NEFA (n = 3−5, mean ± sem).

Previous studies have shown that the ability of CAV1^−/−^ mice to respond to the acute activation of lipolysis by catecholamines is blunted, and that adipocytes from CAV1^−/−^ mice show impaired prolipolytic PKA signalling and PLIN1a phosphorylation, despite elevated levels of PKA activity against other substrates and normal levels of the ß3-adrenergic receptor [18]. However, studies using different CAV1^−/−^ mouse strains have shown that the genetic background can impact on some consequences of CAV1 deficiency [22], [29], [30]. Consistent with Cohen *et al*. (2004) we found that acute *in vivo* stimulation of lipolysis using the beta-3 adrenergic receptor agonist CL316, 243, in an independent strain of CAV1^−/−^ mice (generated by a different genetic deletion [26]), also failed to elicit phosphorylation of PLIN1a or HSL in adipose tissue (results not shown). Furthermore, in adipose tissue explants from CAV1^−/−^ mice neither isoproterenol, nor the direct elevation of cAMP using a combination of forskolin and IBMX, was able to elicit phosphorylation of HSL by PKA, or the release of NEFAs or glycerol, despite high levels of expression of the PKA catalytic subunit (Fig. 1). As we have previously shown that the phosphorylation of HSL by PKA on specific sites occurs at distinct locations in the cell, and in a sequential and hierarchical manner [27], the lack of response to elevated cAMP suggests a primary defect in the coupling of PKA activity to HSL.

**Figure 2:**
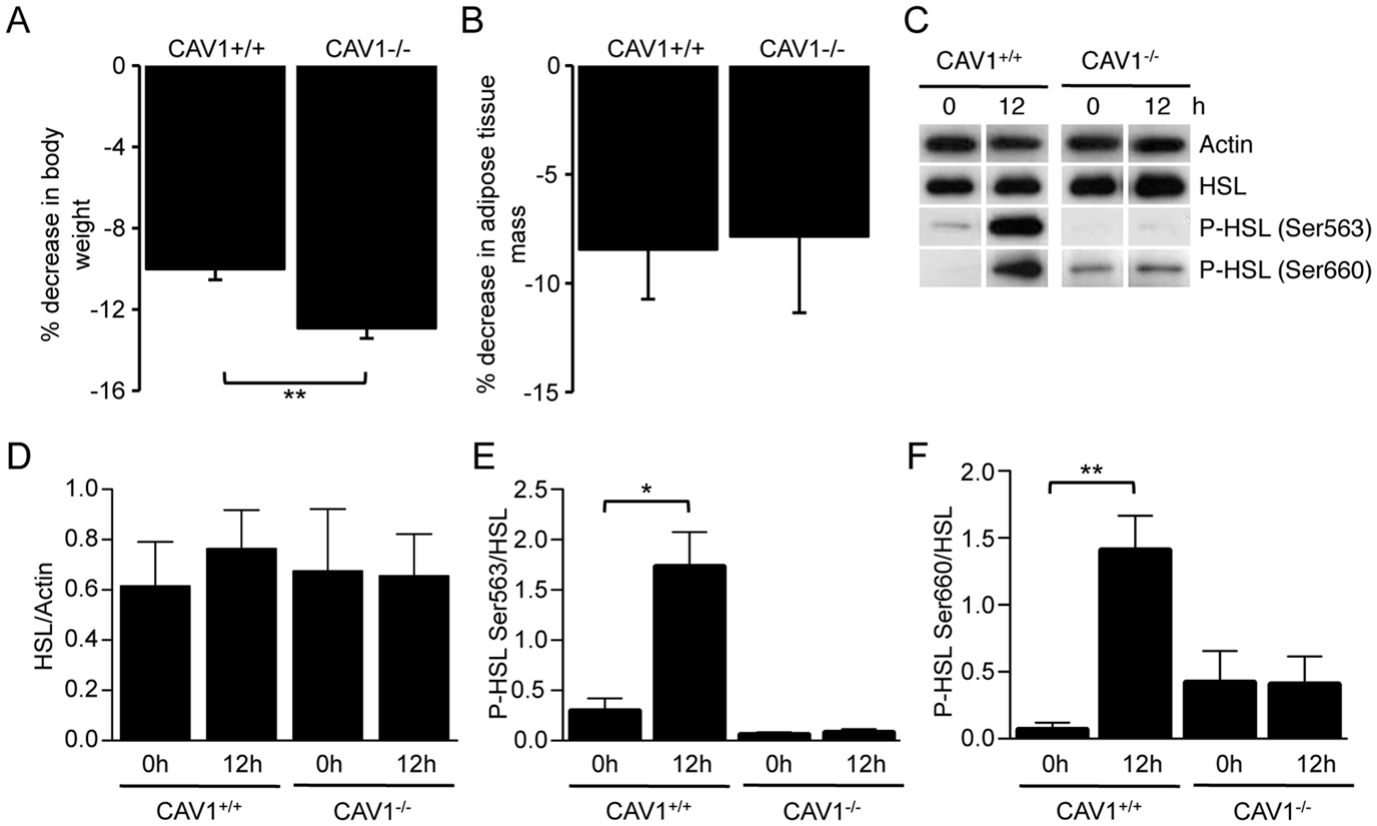
CAV1^−/−^ mice catabolise adipose tissue during fasting despite a block in HSL phosphorylation. CAV1^+/+^ and CAV1^−/−^ mice were weighed prior to and directly following a 24 h fast (**A**). Results are shown as the percentage loss of starting body weight. There was a significantly greater loss of body weight in the CAV1^−/−^ background (n = 3). (**B**) Adipose tissue mass in CAV1^+/+^ and CAV1^−/−^ mice was measured by DXA prior to and following a 24 h fast. Results are shown as the percentage loss of adipose tissue mass relative to starting mass. The reduction in whole body adipose tissue was identical in both backgrounds (n = 5). **C**) The protein level and phosphorylation of HSL in epididymal adipose tissue following a 12 h fast was determined. (**D**) The protein level of HSL was quantified relative to actin. (**E,** **F**). Phosphorylation of HSL on Ser563 and Ser660 was quantified relative to total HSL. Mean ± sem, n = 3. *p<0.05, **p<0.01.

Recent studies have found that fasting results in a decrease in body weight and adipose tissue mass, concomitant with increased serum free fatty acids levels in CAV1^−/−^ mice [21]. Consistent with this we found that after a 24 h fast the percentage weight loss in CAV1^−/−^ mice was actually greater than in CAV1^+/+^ mice (Fig. 2A). Furthermore, the contribution of adipose tissue to weight loss during fasting, as determined by DXA, showed that both CAV1^+/+^ and CAV1^−/−^ mice lose an equivalent percentage of their total body adipose tissue (Fig. 2B), consistent with increased catabolism of adipose tissue regardless of CAV1 expression. However, examination of the phosphorylation status of HSL midway through the fasting period (12 h) demonstrated that while there was significant phosphorylation of HSL on both PKA-targeted sites (Ser563 and Ser660) in CAV1^+/+^ mice, there was no detectable phosphorylation of HSL on either site in the adipose tissue of CAV1^−/−^ mice (Fig. 2C–F). These data suggest that hydrolysis of stored triglycerides during fasting in wild type mice is catalysed by lipid droplet-associated, phosphorylated HSL, whereas the hydrolysis of triglycerides in CAV1^−/−^ mice occurs through a distinct mechanism that does not require HSL phosphorylation by PKA. As phosphorylation of HSL is required for its translocation to the lipid droplet surface [27], these data indicate a general defect in PKA-mediated pro-lipolytic mechanisms in CAV1^−/−^ mice in response to both fasting and to acute adrenergic stimulation.

### Adipose Tissue Macrophage Infiltration and IL-6 Secretion from Adipose Tissue Explants

**Figure 3:**
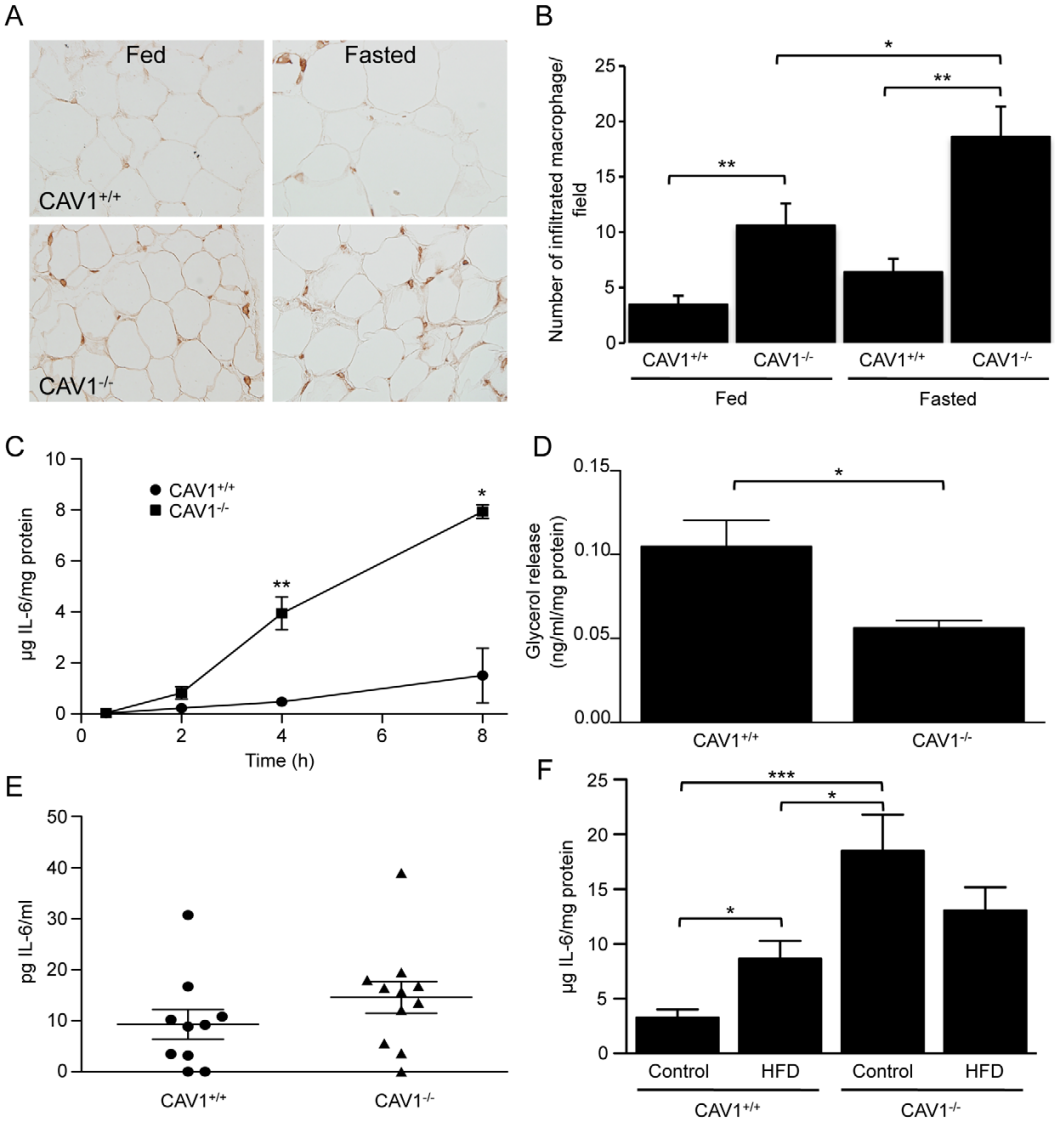
Macrophage infiltration and IL-6 analysis in CAV1^−/−^ adipose tissue. The infiltration of macrophage into adipose tissue and the release of IL-6 was analysed in CAV1^+/+^ and CAV1^−/−^ adipose tissue from fed and 24 h-fasted mice. (**A**) Immunohistochemistry of the macrophage marker protein F4/80 showing macrophage infiltration in CAV1^+/+^ and CAV1^−/−^ adipose tissue from fed and 24 h-fasted mice. (**B**) Quantitation of F4/80-positive infiltrated macrophage (n = 6−8 mice). (**C**) The release of IL-6 from epididymal adipose tissue explants of CAV1^+/+^ and CAV1^−/−^ mice was measured over an 8 h period (mean ± sem, n = 3−6, except 8 h where n = 2). (**D**) The basal release of glycerol from unstimulated explants was determined after 4 h in culture (mean ± sem, n = 5−6). (**E**) Systemic IL-6 levels in serum from CAV1^−/−^ and CAV1^+/+^ mice following a 24 h fast (mean ± sem, n = 10−11 mice). (**F**) The release of mIL-6 over 4 h from epididymal adipose tissue explants of CAV1^+/+^ and CAV1^−/−^ mice maintained on a Control or high fat diet (HFD) (mean ± sem, n = 5−6). *p<0.05, **p<0.01, ***p<0.001.

The observed loss of adipose tissue during fasting in CAV1^−/−^ mice suggested that triglycerides were being hydrolysed by an alternative, PKA-independent mechanism. Cytokines, such as TNF and IL-6 have been shown to stimulate lipolysis, in part through the degradation of the lipid droplet coat protein, PLIN1a [31], [32]. We considered the possibility that cytokines produced by adipose tissue could be a contributing factor to the regulation of lipolysis in CAV1^−/−^ mice. Consistent with previous studies we found an increased infiltration of macrophages into the adipose tissue of CAV1^−/−^ mice under fed conditions (Fig. 3A–B) [21], [33]. Furthermore, fasting stimulated a greater infiltration of macrophage into CAV1^−/−^ tissue compared to CAV1^+/+^ tissue (Fig. 3A–B). To examine cytokine production by adipose tissue we used a multiplex FACS assay to screen the levels of a variety of cytokines in the culture media of adipose tissue explants from CAV1^−/−^ and CAV1^+/+^ mice. While most cytokines measured were undetectable in the media or showed no difference between CAV1^+/+^ and CAV1^−/−^ mice (including TNF, IL-10, MCP-1, IFNgamma and IL12p70, results not shown), we found that the secretion of IL-6 from CAV1^−/−^ adipose tissue was significantly higher than from CAV1^+/+^ tissue (Fig. 3C). As IL-6 has previously been suggested to up-regulate lipolysis in adipose tissue we hypothesized that the increased secretion of IL-6 could underlie the catabolism of adipose tissue during fasting. However, when we examined the basal rate of lipolysis in explants of adipose tissue over a 4 h period we found a significant suppression of basal glycerol release despite the high levels of IL-6 being secreted into the media (Fig. 3D). Furthermore, systemically we found no difference between CAV1^−/−^ and CAV1^+/+^ mice IL-6 serum levels following a 24 h fast (Fig. 3E), and IL-6 failed to elicit an increase in glycerol release from 3T3-L1 adipocytes whereas another pro-inflammatory cytokine, TNF, caused a small but significant increase over 24 h consistent with previous studies (results not shown) [34]. Finally, as pro-inflammatory cytokine production by adipose tissue is a hallmark of obesity [35], we analysed the production of IL-6 by adipose tissue explants from CAV1^+/+^ and CAV1^−/−^ mice that had been maintained on a high-fat diet (HFD) or a control diet for 12 weeks. Consistent with previously published data, CAV1^+/+^ mice showed a significant increase in weight and adipose tissue mass on the HFD compared to the control diet, whereas CAV1^−/−^ mice were resistant to diet-induced weight gain [21], [22], [23]. When we examined the secretion of IL-6 from adipose tissue explants we found a significant increase in CAV1^+/+^ mice on the HFD, and from CAV1^−/−^ mice on either diet (Fig. 3F). The release of IL-6 from control diet CAV1^−/−^ adipose tissue explants was significantly greater than from adipose tissue explants obtained from the high fat fed CAV1^+/+^ mice. Furthermore, the combined effect of CAV1 deficiency and high fat feeding did not cause a significant increase in IL-6 release relative to either condition alone, suggesting that the absence of CAV1 alone is sufficient to saturate the inflammatory response capacity. Together these data indicate that while there is an increased capacity to produce IL-6 in the adipose tissue of CAV1^−/−^ mice, this is unlikely to directly regulate the loss of adipose tissue during fasting. In addition, it suggests that increased secretion of IL-6 characterizes different types of adipose tissue metabolic stress, i.e. diet induced obesity and CAV1 deficiency.

### PLIN1a Protein Levels Decrease during Fasting and in ex vivo Explant Cultures of CAV1^−/−^ Adipose Tissue, and in Cultured Adipose Tissue Explants from Obese CAV1^+/+^ Mice

**Figure 4:**
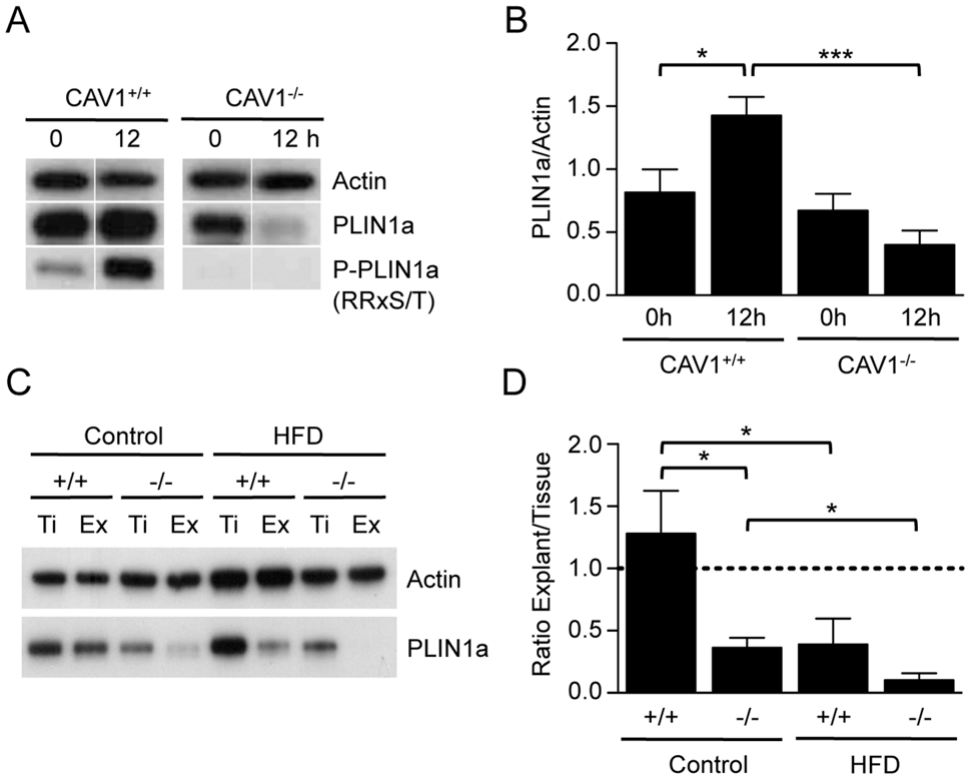
Expression and phosphorylation of PLIN1a during fasting and *ex vivo* culture. The protein levels and phosphorylation of PLIN1a in epididymal adipose tissue from fed mice and following a 12 h fast (**A, B**). The expression of PLIN1a was quantified relative to actin. Phosphorylation of PLIN1a was determined using an antibody to phosphorylated PKA-substrate (RRxS/T). (**C, D**) The protein level of PLIN1a was compared between tissue (Ti) and explants of the same tissue following *ex vivo* culture for 4 h (Ex). Adipose tissue was analysed from mice maintained on a Control or high fat (HFD) diet for 12 weeks. The expression of PLIN1a in explants was quantified relative to tissue (mean ± sem, n = 3). *p<0.05, ***p<0.001.

In addition to acting as an adaptor protein for prolipolytic proteins including HSL, the lipid droplet protein PLIN1a also acts as a barrier to basal lipolysis [36]. Under certain conditions, including elevated levels of proinflammatory cytokines, increased lipolysis can be associated with a selective reduction in PLIN1a levels [31], [32]. We therefore examined the expression level of PLIN1a in adipose tissue after fasting for 12 h. While both CAV1^+/+^ and CAV1^−/−^ mice expressed very similar levels of PLIN1a under fed conditions, following a 12 h fast CAV1^+/+^ adipose tissue showed a slight increase in PLIN1a expression, whereas the levels of PLIN1a were significantly reduced in CAV1^−/−^ adipose tissue relative to CAV1^+/+^ tissue (Fig. 4A,B). As PLIN1a has a reported half-life of ∼70 h [32] this suggests an active mechanism promoting PLIN1a degradation. In response to the activation of lipolysis PLIN1a is hyper-phosphorylated by PKA, resulting in the appearance of a prominent phosphoprotein that can be readily identified using a pan-PKA substrate antibody (RRxS/T). In CAV1^+/+^ adipose tissue PLIN1a was robustly phosphorylated after 12 h fasting (Fig. 4A). In contrast, in CAV1^−/−^ adipose tissue phosphorylation of PLIN1a was undetectable after 12 h fasting.

To determine whether the loss of PLIN1a observed *in vivo* during fasting could be recapitulated in an *ex vivo* situation we cultured adipose tissue explants from CAV1^+/+^ and CAV1^−/−^ mice for 4 h in DMEM supplemented with 0.1% fatty acid-free bovine serum albumin. While the levels of PLIN1a remained constant in CAV1^+/+^ explants, we found that PLIN1a levels were significantly reduced in CAV1^−/−^ explants relative to the starting levels in the tissue (Fig. 4C–D). Interestingly, we found that adipose tissue from mice maintained on a high fat diet for 12 weeks also showed a significant decrease in PLIN1a levels following *ex vivo* culture, irrespective of CAV1 expression. These data show that while PKA signalling is blunted in CAV1^−/−^ adipose tissue there are significant changes in the regulation of adipocytes and lipid droplet structural proteins, specifically PLIN1a, that could underlie adipose tissue catabolism in the absence of CAV1.

### Adipose Tissue Fibrosis and Susceptibility to Cell Death

Finally, previous histological analysis of adipose tissue from CAV1^−/−^ mice on a high fat diet had suggested that in addition to increased macrophage infiltration, there was also increased cell death in the adipose tissue of CAV1^−/−^ from mice, which correlated with a loss of PLIN1a labelling [21]. We therefore considered the possibility that increased adipocyte cell death could be a contributing factor to adipose tissue loss during fasting.

**Figure 5:**
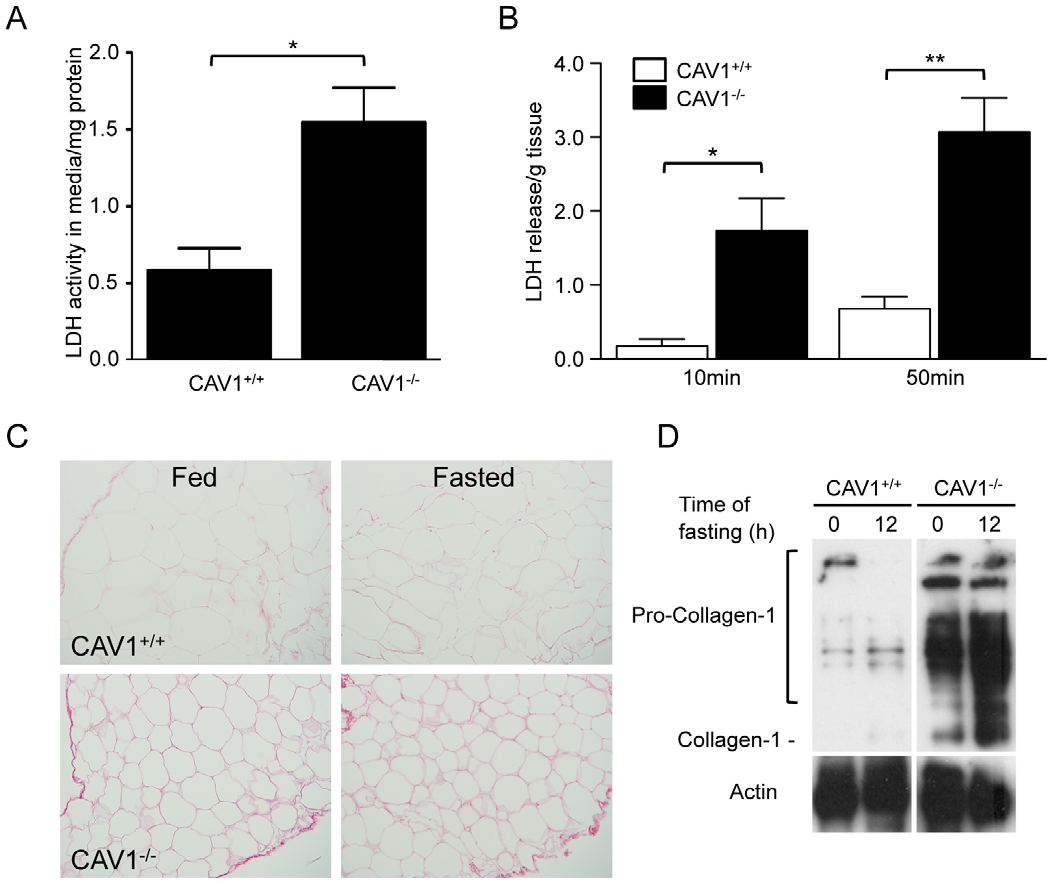
Fibrosis and cell death characterises adipose tissue from CAV1^−/−^ mice. The release of LDH and the deposition of collagen was analysed in CAV1^+/+^ and CAV1^−/−^ adipose tissue. (**A**) LDH activity in the culture media of epididymal adipose tissue explants after 4 h *in vitro* (mean ± sem, n = 5−6). (**B**) Release of LDH from adipose tissue explants during collagenase digestion (mean ± sem, n = 3). (**C**) Sirius red staining for collagen in paraffin-embedded sections of CAV1^+/+^ and CAV1^−/−^ adipose tissue from fed and 24 h-fasted mice. (**D**) Protein levels of pro-collagen in CAV1^+/+^ and CAV1^−/−^ adipose tissue from fed and 12 h fasted mice determined by Western blotting of whole tissue lysates. *p<0.05, **p<0.01.

To gain further insights into adipose tissue stability we examined the release of lactate dehydrogenase (LDH), a well-known marker of acute or chronic tissue damage. Consistent with decreased integrity of the plasma membrane in adipose tissue explants from CAV1^−/−^ mice we found greater amounts of LDH were released into the medium of CAV1^−/−^ explants as compared to CAV1^+/+^ tissue after 4 h of culture (Fig. 5A). Intriguingly, this difference was further exacerbated by treatment with collagenase, resulting in a rapid and marked increase in the rate of LDH release by CAV1^−/−^ explants compared to CAV1^+/+^ tissue (Fig. 5B). This effect was so marked as to render CAV1^−/−^ primary adipocytes, isolated by collagenase treatment, incapable of being cultured under identical conditions to CAV1^+/+^ cells.

Intriguingly, genetic loss of adipose collagen has been reported to increase caveola density in adipocytes [37]. We therefore examined collagen deposition in the adipose tissue of fed and fasted CAV1^+/+^ and CAV1^−/−^ mice. CAV1^−/−^ adipose tissue showed increased collagen deposition (Fig. 5C) and a higher level of pro-collagen in the tissue (Fig. 5D). This was accompanied by a marked difference in the physical properties of the tissue; the adipose tissue of CAV1^−/−^ mice was significantly less flexible suggesting a significantly modified extracellular environment (SM and MAFR, empirical observations). Together these data show an increased fragility of adipose tissue of CAV1^−/−^ mice *in vitro*. The strikingly increased susceptibility to cell lysis after collagen digestion suggests that increased collagen deposition in the CAV1^−/−^ tissue may serve to partially compensate for increased fragility of adipocytes.

## Discussion

In this study we have examined the effect of CAV1 deficiency on mouse adipose tissue as a model of human lipodystrophy. CAV1-deficient mice are resistant to diet-induced obesity and have defects in adipose tissue lipolysis [18], [21], [22], [23], while humans with caveolin-1 mutations show a severe lipodystrophy [24]. We show that CAV1 deficiency significantly blunts the canonical PKA-mediated prolipolytic pathways in adipocytes but alternative molecular mechanisms allow adipose tissue lipid mobilization in response to fasting. Adipose tissue from CAV1^−/−^ mice shows increased cell lysis when isolated and cultured as tissue explants, and this susceptibility to damage is even more pronounced upon removal of the collagen matrix. These studies suggest that the previously proposed roles of CAV1 in mechano-protection [38] and modulation of the extracellular environment in response to mechanical stimuli [39] might underlie the lipodystrophic phenotype of CAV1^−/−^ mice and patients.

The striking phenotypic changes in the adipose tissue of CAV1^−/−^ mice include an increase in macrophage infiltration, increased capacity for IL-6 production and secretion *in vitro*, increased collagen deposition and loss of PLIN1a protein. Adipose tissue is one of the major tissue sources of inflammatory cytokines including TNF and IL-6 [40], [41], the circulating levels of which correlate directly with adiposity [42]. As previous studies had shown that IL-6 could stimulate lipolysis in humans [43] and directly activate lipolysis in porcine adipocytes [44], where it also results in a down-regulation of PLIN1a, we considered the possibility that IL-6 directly stimulates lipolysis in the adipose tissue of CAV1^−/−^ mice. However, we found no significant change in systemic IL-6 during fasting, recombinant IL-6 failed to elicit an increase in glycerol release in 3T3-L1 adipocytes, and adipose tissue explants of CAV1^−/−^ mice had suppressed basal lipolysis despite the high levels of IL-6 present in the culture media. We therefore consider it unlikely that IL-6 is directly responsible for adipose tissue lipid metabolism during fasting in CAV1^−/−^ mice. However, in several pathological conditions associated with increased fibrosis, such as systemic sclerosis [45], IL-6 can act as a profibrotic cytokine, suggesting the increase in collagen deposition observed in CAV1^−/−^ adipose tissue could be related to the elevated levels of IL-6.

Adipose tissue lipolysis is catalysed by a sequential series of lipase enzymes, the activities of which are tightly controlled by a variety of mechanisms including accessory proteins/co-activators and post-translational modifications (reviewed by Zechner et al., 2012 [46]). While we have restricted our analysis of lipolysis in CAV1^−/−^ mice to HSL, which predominantly catalyses the conversion of diacylglycerol (DAG) to monoacylglycerol, the loss of PLIN1a could potentially have a direct effect upon the activity of the predominant TAG to DAG lipase, adipose triglyceride lipase (ATGL). ATGL activity depends upon a co-activator protein, CGI-58, which binds to PLIN1a in unstimulated cells, thereby preventing ATGL activity. During normal activation of lipolysis the phosphorylation of PLIN1a by PKA results in its dissociation from CGI-58, freeing CGI-58 to interact with and activate ATGL. One interesting possibility that requires further investigation is that ATGL activity could be directly elevated by the reduction in PLIN1a levels observed in CAV1^−/−^ adipose tissue during fasting, allowing partial mobilisation of TAG-derived fatty acids and DAG.

In addition to an inflammatory response and macrophage infiltration, CAV1^−/−^ adipose tissue was characterized by increased fragility of the adipocytes, as shown by susceptibility to cell lysis, and the remodelling of the extracellular matrix (ECM), as indicated by increased collagen deposition. Recent studies have implicated caveolae in protection of the plasma membrane in response to membrane stress [38] and shown a role of CAV1 in remodelling of the extracellular environment [39]. Caveolins work together with the cytoplasmic cavin complex to regulate caveolar formation [47], [48], [49], [50], [51] and Cavin-1/PTRF has been linked to regulation of collagen transcription mediated by BFCOL1 in fibroblasts [52]. Membrane force causes flattening of caveolae and release of the cavin complex into the cytosol [38] providing a potential link between surface forces and ECM synthesis. The collagen matrix, including collagen VI [37], surrounding adipocytes provides crucial mechanical support to the adipocyte, which is subjected to considerable forces. The extracellular matrix must be remodelled to allow adipocyte expansion and it has been shown that changes in the matrix can directly contribute to metabolic dysregulation, including an inability to change adipocyte mass in response to demand [37]. It is therefore tempting to speculate that dysregulation of ECM deposition in response to membrane forces required for normal adipose tissue function is perturbed in the absence of caveolae. In addition, the inability of the adipocyte membrane to flatten caveolae in response to increased membrane tension, or to buffer lipolytic flux, could lead to membrane damage. The changes in the properties of the tissue are emphasized by the inability to isolate viable primary adipocytes from the adipose tissue of CAV1^−/−^ mice by standard collagenase digestion techniques, despite this being a well-established procedure for culture of primary adipocytes. Release from the collagen matrix is sufficient to trigger cell lysis.

Recent work has shown that *in vitro-*differentiated adipocytes derived from CAV1^−/−^ MEFs show a higher sensitivity to lipolytic flux than CAV1^+/+^ MEF-derived adipocytes [20]. In addition, previous studies have shown that in adipocytes TAG can be synthesized from exogenously added fatty acids within a subpopulation of cell surface caveolae, suggested to protect adipocytes from potential membrane disruption due to saponification [53]. Together with the present study, these results suggest that one function of CAV1 in normal adipose tissue is to support the mobilization of fatty acids by buffering the plasma membrane against the detergent effects of large quantities of NEFAs, and against shear stresses generated by the large lipid droplets, which occupy the bulk of the cytoplasm. Increased collagen deposition in the CAV1^−/−^ tissue would partially compensate for this *in vivo.* This model suggests that the proposed role for caveolae in adrenergic signaling could be coupled to a protective effect monitoring the flux of NEFAs. As *in vitro-*differentiated adipocytes derived from CAV1^−/−^ MEFs do not show the loss of prolipolytic signalling observed in adipose tissue of CAV1^−/−^ mice [20] this suggests that tissue specific perturbations underlie the dysfunction of adipose tissue in CAV1-related lipodystrophies. This model provides an attractive testable hypothesis for understanding the role of caveolae in normal adipose tissue and dysfunction in disease.
